# Continued Participation of Israeli Adolescents in Online Sports Programs during the COVID-19 Pandemic Is Associated with Higher Resilience

**DOI:** 10.3390/ijerph18084386

**Published:** 2021-04-20

**Authors:** Keren Constantini, Irit Markus, Naomi Epel, Ronit Jakobovich, Yftach Gepner, Shahar Lev-Ari

**Affiliations:** 1 Department of Epidemiology and Preventive Medicine, School of Public Health, Sackler Faculty of Medicine, and Sylvan Adams Sports Institute, Tel-Aviv University, Tel-Aviv 6997801, Israel; kconstantini@mail.tau.ac.il (K.C.); iritmarkus@mail.tau.ac.il (I.M.); 2 Department of Health Promotion, School of Public Health, Sackler Faculty of Medicine, Tel-Aviv University, Tel-Aviv 6997801, Israel; epelsh@gmail.com (N.E.); jakobovich.ronit@gmail.com (R.J.)

**Keywords:** adolescents, participation in physical activity, resilience, online programs, wellbeing

## Abstract

*Background:* Coronavirus disease 2019 (COVID-19) has forced adolescents to adapt rapidly to a new reality of physical and social distancing, while introducing a range of new sources of stress and adversity. Our primary aim was to study the relationship between adolescents’ resilience and their participation in online sports programs during the COVID-19 pandemic lockdown period. Our secondary aims were to assess the associations between the organized sports programs’ determinants and resilience. *Methods:* Online surveys designed to examine resilience, lifestyle, psychosocial health and characteristics of the organized sports programs were administered to 473 adolescents who were enrolled in organized sports programs before the COVID-19 pandemic. *Results:* Adolescents who continued to participate in online structured programs during the lockdown period were significantly more resilient and physically active, had higher self-related health, satisfaction with life, and ability to cope during the pandemic, compared to those who did not participate. Relationships with the adult instructor and levels of physical activity were the most important factors of the programs that were associated with resilience. *Conclusions:* Participation of adolescents in sports programs is an important resource associated with higher levels of resilience. Youth programs should continue their activities during globally challenging times, such as the COVID-19 pandemic.

## 1. Introduction

The severe acute respiratory syndrome coronavirus-2 (SARS-CoV-2) outbreak and associated coronavirus disease 2019 (COVID-19) have forced a new and highly dynamic reality on the entire world population. This pandemic has exposed adolescents to many new sources of stress, such as exposure to excessive media (*infodemic*), pessimistic news, and adults’ stress and anxiety in their household and community [1,2]. Furthermore, the new reality was accompanied by constant changes to daily routines, physical distancing from family and friends, and in some cases, limited ability to perform regular physical activity. The psychological effects on adolescent mental health as a result of quarantine and social distancing, as implemented in numerous countries around the globe, may be long-lasting and manifest as symptoms related to heightened psychological distress, such as depression, anxiety, confusion, and emotional disturbance [3,4]. Such negative outcomes are reduced in adolescents who are involved in organized sports programs [5,6,7,8]. However, with the restrictions imposed by the new COVID-19 reality on structured activities, specifically those with in-person activities, the benefits of continuing these sports programs in a virtual format remain unknown, despite having a potentially positive effect on coping mechanisms (i.e., resilience) in adolescents during the pandemic lockdown.

Resilience is the ability to adapt successfully in the face of stress and adversity. This complex, dynamic, and the individualized process involves both internal strength (assets) and external resources [9,10,11]. A “one size fits all” model cannot be applied to predicting how an individual will respond to stressful situations because people are inherently different, and resilience is an evolving trait that is context-dependent. As such, responses and coping mechanisms for dealing with adversity vary from person to person [12]. Common traits that promote positive outcomes are associated with resilience [9]. The most relevant internal factors include high self-esteem and confidence in one’s strengths and abilities [13,14,15], while the external resources relate to adult mentors, opportunity structures (i.e., settings, or structures that “shape the individual’s capacity to experience resilience when facing adversity”) [16], social networks/support, and having a number of friends. All these factors have been identified as important for managing one’s immediate surroundings and coping with adversity [17,18,19].

One approach for developing resilience among youth is through organized activities such as sports programs [5,6,9,20]. The social support of organized programs provides a framework and platform for individual growth and initiative, thus promoting the development of both internal and external resources that enhance resilience in a safe environment [9]. In addition, maintaining regular physical activity is an important internal resource that can enhance resilience and improve physical and mental health during the SARS-CoV-2 crisis [8,21,22]. It, therefore, follows that virtual organized sports programs may be able to reduce anxiety and depression symptoms and enhance resilience by providing the opportunity to maintain regular physical activity at home while also developing friendships with peers and close relationships with an adult instructor [8,9]. While several sports programs and youth movements have continued to take place during the lockdown period via online platforms (e.g., Five Fingers sports educational organization), many other programs (e.g., the Scouts movement) have discontinued their activities. To the best of our knowledge, no data exist regarding the association between resilience and continued participation in online sports programs among adolescents *during* the COVID-19 pandemic and lockdown.

This study’s primary aim was to evaluate the relationship between participation in an online, structured sports program and the resilience of adolescents during the COVID-19 pandemic lockdown period. We hypothesized that adolescents who continued to participate in online versions of their programs would be more resilient than those who ceased to participate in the programs’ activities. Our secondary aims were to assess the correlations between resilience and program variables. We hypothesized that resilience would be related to structured physical activity and social support.

## 2. Methods & Materials

This cross-sectional study was conducted in Israel between 10 April, 2020 and 5 May, 2020. A social web-based sample of the adolescent population of Israel was recruited to participate in the study to assess adolescents’ resilience, lifestyle, psychosocial health, and characteristics of the organized sports program in which they participated during the outbreak of COVID-19. The final sample comprised 473 Israeli adolescents between the ages of 16–18 years, participating in sports programs in 62 municipalities.

### 2.1. Study Sample and Data Collection

The sample was planned using G-Suite as an online survey tool for the quick and effective distribution of an online questionnaire. We used the self-selection online survey method of nonprobability sampling [23] to recruit participants through social networks, and asked Israeli adolescents to answer the survey. The rationale for distributing questionnaires via social networks was related to the lockdown strategy that was implemented at that time, and restricted most of the population from leaving their homes and meeting other people. The survey was distributed to the public in two stages and using three main social media platforms: Facebook, WhatsApp, and Instagram. During the first stage, intensive sampling was conducted through the social networks and social media platforms via sports education organizations/programs (e.g., Five Fingers sports educational organization) and municipality networks. During the second stage, snowball sampling was conducted to reach broader circles within the Israel communities [24], followed by additional dissemination through diverse community circles in the adolescents’ community forums and Facebook page circles. Inclusion criteria included adolescents between the ages of 16–19 years who consented to participate and whose parents approved consent. The study was approved by the Tel-Aviv University Institutional Ethics Committee (Approval no. 20/088, April 2020). Adolescents and parents approved participation in the survey. Participants could withdraw from the survey at any given moment, with no need for justification.

### 2.2. Survey Tools

A questionnaire was developed for this study after introducing the survey and requesting informed consent of the participant and their parents. The questionnaire inquired about the demographic background of the adolescent. The questionnaire included four sections concerning resilience, lifestyle, psychosocial variables, and the sporting organization’s characteristics.

*Resilience:* The Connor–Davidson resilience scale (CD-RISC-10), which has been previously validated for this population, was used to assess resilience [25]. The ten-items are phrased so that a higher endorsement of a statement indicates higher resilience (0 = not at all true, 1 = rarely true, 2 = sometimes true, 3 = often true, and 4 = true nearly all the time). People with lower-resilience categories tend to rate individual items in the 0–2 range, those with medium resilience tend to rate items as 3, and those with higher resilience tend to rate items as 4. A total of ten items were presented; CD-RISC scores range from 0 to 40.

*Lifestyle and Psychosocial variables:* health behavior assessments and psychosocial questions were adopted from The Health Behaviors in School-aged Children (HBSC) survey for adolescents validated in 50 countries, including Israel [26,27]. The health behavior assessments included questions on physical activity (number of times per week of at least 60 min or more of moderate-to-vigorous physical activity), sleep (average hours per day) and nutrition (eating a healthy breakfast). Psychosocial health queries included questions about self-related health, satisfaction with life, mood, and social support. The validated Rosenberg Self-Esteem (RSE) scale was used to assess self-esteem [28].

*Characteristics of the organized sports program:* This questionnaire was developed for this research with organized sports program coaches and two of the study authors (R.Y and O.E), both experts in adolescents’ health promotion. Twenty questions queried about the organization’s type and number of activities per week, duration of participation, components of the activities, motivation to participate, relationship with the adult instructor or guide, and the continued function of the organization during the COVID-19 pandemic (Supplementary S1). We used a Likert scale, ranging from 1 (not important at all) to 5 (very important) to assess which program activities (content of physical exercise, session initiation and closure discussions, relationship with the instructor or guide, relationships with friends in the group, and program special events) the participants perceived as important. A pilot testing and reliability analysis were conducted by performing a pilot study of 30 participants in a convenience sample. The participants were asked to provide feedback about the questionnaire, including length of time needed, wording, the relevance of questions, etc. Changes were made to adapt the questionnaire for adolescents. The Cronbach’s alpha reliability of program activities (questions 12–16, Supplementary S1) was 0.93, and test–retest reliability was 0.89, indicating a very high validity score.

For the entire cross-sectional study, the validity of questionnaires was tested via Pearson’s correlation coefficient, examining linear relationships between consecutive variables. Reliability was tested via Cronbach’s alpha measure. For resilience, the score was 0.814, for self-esteem, the score was 0.87, and for the program activities, the score was 0.87.

### 2.3. Analytic Approach

Descriptive statistics present the number of adolescents with percentages for categorical variables and means and standard deviations for continuous variables. Independent (?) T-tests for interval data and chi-squared tests for nominal level data were used to compare adolescents who continued to participate in online sports programs during the lockdown period to those who did not. A sensitivity analysis with gender as a covariate was conducted due to differences between groups at baseline. Sample size calculation was done using WinPEPI software (version 11.65,(PEPI-for-Windows, Brixton Health, London, UK) and was based on the expected change of 1.5 in resilience total test score, the main variable of this study. A required sample size of 424 participants was calculated to achieve an alpha of 5% and 80% power.

Multiple regression analyses using a stepwise approach were used to determine the associations between the characteristics of the organized sports programs and resilience as the primary outcome. Variables were further adjusted for age, gender, and socioeconomic status. SPSS 25 (IBM, New York, NY, USA) software was used for statistical analyses. Associations with a *p*-value < 0.05 were considered to be statistically significant.

## 3. Results

### 3.1. Baseline Demographics Characteristics

A total of 473 adolescents participated in this cross-sectional study. Table 1 presents characteristics of the study population in adolescents who continued to participate in online sports programs during the lockdown period (*n* = 333) and those who did not (*n* = 140). Both groups had a mean age of 17 (mostly in 10th or 11th grade), and the vast majority (over 90%) were born in Israel. There was a larger percentage of males in the group that continued to participate in virtual activities than in the group whose program did not function during lockdown (69% vs. 51%, respectively, *p* = 0.001). None of the other variables exhibited a significant difference between the groups.

Scores for resilience, lifestyle, and psychosocial variables across the two groups are shown in Table 2. Adolescents who continued to participate in sports group meetings reported significantly higher levels of resilience (31.0 ± 5.3 and 29.3 ± 6.1, respectively, *p* = 0.005) than those who did not.

### 3.2. Relationship between Organized Sports Programs Components and Resilience—Univariate and Multivariate Analysis.

Adolescents who continued to participate in their programs’ online activities reported higher levels of physical activity (242.5 ± 101.03 min/week compared to 191.1 ± 145 min/week, *p* < 0.001). Yet, only 10.6% of the participants in both groups adhered to recommended physical activity guidelines for children and adolescents (ages 6 to 17 years) to do 60 min or more of moderate-to-vigorous physical activity daily, and only 22% of the adolescents were active for 60 min/day 6 days/week. Since the mean age of adolescents in our sample was 17.4 ± 0.8, we also assessed adherence to recommended physical activity guidelines for adolescents above 17 years (>150 min/week). Using this criterion, improved adherence to recommended guidelines was found in adolescents who continued to participate compared to those who did not (83.1% vs. 53.6%*,* χ^2^ = 39.85, *p* < 0.001). Both groups had a similar average of 8 h of sleep a night and did not differ in breakfast consumption habits.

Overall, adolescents who continued to participate in sports group meetings (online) reported higher psychosocial health: 78% reported being in excellent health compared to 64% in the nonparticipating group (*p* = 0.006). They were also happier than nonparticipants (satisfaction with life score of 7.0 and 6.6, respectively, *p* = 0.02) and tended to have higher morale (*p* = 0.09). There was no significant difference in self-esteem between the groups. The majority (>80%) of adolescents in both groups reported that they had three or more close friends, with a higher rate in the nonparticipating group. Loneliness levels were similar between the groups.

In a sensitivity analysis, adjusted for gender, both male and female adolescents who continued to participate in the programs’ online activities reported higher levels of physical activity and better adherence to recommended (>150 min/week) physical activity guidelines for adolescents (male *p* < 0.001; female *p* = 0.029). Yet, in satisfaction with life and self-related health, a significant difference was found between male adolescents, who continued to participate compared to those who did not (*p* = 0.003, *p* = 0.001, respectively), while for women no difference between the groups was found (*p* = 0.892, *p* = 0.960). In contrast, female adolescents who continued to participate reported less loneliness compared to those who did not (*p* = 0.028), while no difference between the groups was found in loneliness levels of male adolescents (*p* = 0.865).

Comparisons between the groups revealed that adolescents who continued to participate in sports group meetings reported significantly higher levels of feeling able to cope well during the COVID-19 pandemic (*p* = 0.03). Most (84%) reported that continued participation helped them improve their ability to cope with pandemic-related negative feelings (χ^2^_(1)_ = 67.0, *p* < 0.001) compared to nonparticipants.

Our next step was to evaluate how the sports program characteristics were perceived by the participants. Adolescents who continued to participate in online sessions of their sports group meetings during the lockdown period, perceived the program components (content of physical exercise, session initiation and session closure discussions, relationships with friends in the group, relationship with the group’s adult instructor, and special program events) as more meaningful than those who did not continue to participate during the lockdown (*p* < 0.001, for all program components, Table 3).

Correlations between program components were found to be significant (*p <* 0.01 for all components), with a medium effect size ranging between *r* = 0.287 to *r* = 0.535 (Table 4). The correlation was highest between “relationships with friends in the group” and “relationship with the group’s adult instructor” variables (r = 0.535, *p <* 0.01).

Analysis by a multivariate model indicated that a positive relationship with the group’s adult instructor was the most important factor associated with resilience (*p* < 0.001), while other variables in the online sports program (the content of physical exercise, session initiation and session closure discussions, relationships with friends in the group, and program special events) did not reach statistical significance. Associations were controlled for age, gender, and socioeconomic status (Table 5).

## 4. Discussion

To the best of our knowledge, this is the first study to evaluate the relationship between participation in online organized sports programs and resilience in adolescents during the COVID-19 pandemic. Our findings suggest that even under conditions of quarantine and social distancing, adolescents who continued to participate in online versions of their existing sports programs were more resilient and physically active, had higher self-related health levels, and expressed greater satisfaction with life, higher morale, and improved ability to cope during the pandemic compared to those who did not continue to take part in such activities. Our multivariate analysis showed that physical activity and relationship with the coach were positively associated with resilience.

During the COVID-19 pandemic, self-quarantine and lockdown periods were not uncommon practices implemented by governments in many countries. Although these new measures were designed to preserve physical health, they also introduced a new challenging reality and new stress sources [3,29]. For adolescents, not only schooling came to a halt, but extracurricular activities that help develop and enhance resilience factors, such as social networks and social support, were also curtailed. The pandemic limited or completely removed these resilience enhancers from the lives of many adolescents at a time when they were perhaps most needed. While longitudinal data regarding the effects of this pandemic on resilience in adolescents are not yet available, it would be reasonable to assume that overall resilience would be negatively impacted by this extreme situation [29,30]. With that, a recent study showed that ~40% of Chinese adolescents experienced symptoms of depression and anxiety during the COVID-19 pandemic [31]. The authors of that study [31] emphasized the importance of increasing governmental bodies’ awareness of the negative psychological outcomes of the pandemic on adolescents. Our results provide a practical tool for combating unfavorable psychological health outcomes, as they indicate that continued involvement and participation in organized sports programs during the COVID-19 pandemic was related to higher levels of resilience (Table 2). In addition, adolescents, who continued to participate in their online structured activities, reported that the program helped them cope well during the pandemic lockdown (Table 2). These findings highlight the importance of sports programs as a social support framework that is associated with adolescents’ resilience, particularly during adverse situations, such as the COVID-19 pandemic.

Adolescents usually conduct their daily physical activity (e.g., commuting, exercising and playing) outdoors at school, sports facilities, playgrounds and parks. Conversely, most of their sedentary time is spent at home. Recent studies suggested that children are less active and more sedentary on unstructured or non-school days and that their levels of sedentary screen time increased at the expense of physical activity during COVID-19 lockdown [32,33]. Our results show that adolescents who continued to participate in programs’ online activities reported higher levels of physical activity (Table 2). Current recommendations for children and adolescents aged 6 through 17 years are 60 min of moderate-to-vigorous intensity physical activity each day [34]. Our findings show that 22% of the adolescents were active for 60 min/day 6 days/week and 11% for 7 days/week. These data are in line with previous large studies [35]. In addition, since most of the participants in this study were above 17 years, we also used recommended guidelines for the general population of >150 min/week. When these guidelines are considerd, improved adherence to recommended guidelines was found in adolescents who continued to participate compared to those who did not (83.1% vs. 53.6%, respectively, *p* < 0.001)

This finding indicates that, while adolescents perform some physical activity, even when their sports group is not active during the lockdown period, online programs may encourage adolescents to maintain recommended physical activity. The results were consistent with previous studies. Sundar et al. [36] found that Web-based interventions of physical activity, conducted within organized sports programs, supported adolescents’ motivation to participate in and maintain physical activity through providing fun, social interactions with friends, and the experience of mastering an activity. Our finding emphasizes the importance of developing online activities in addition to traditional (face-to-face) activities of sports programs to support adolescents’ motivation to maintain recommend physical activity guidelines.

The psychological effects of quarantine can be long-lasting and lead to symptoms associated with heightened psychological distress such as depression, confusion, and emotional disturbance [3]. These potentially negative outcomes have been shown to be reduced in adolescents who are more physically active [5,7,8], and the physical and mental health benefits of remaining physically active during the pandemic are now appreciated [37]. Although our study was cross-sectional in nature, and therefore, cause-and-effect relationships cannot be determined, our multivariate analysis (Table 5) found an association between resilience and physical activity. Our findings suggest that routine physical activity supports adolescents’ resilience during the pandemic lockdown periods.

Adolescence is a challenging period of life, with over 10% of youth experiencing symptoms of anxiety and depression. The absence of external resilience-promoting resources, such as support from friends and social programs, and the loss of feelings of security and safety during times of emergency [29], such as the COVID-19 pandemic, are likely to increase the susceptibility to symptoms of anxiety and depression among adolescents [28]. This has also been shown to be true during the COVID-19 pandemic [30]. Our results show that adolescents who continued to participate in online sports programs rated their morale and satisfaction with life at a higher level than those who did not continue to take part in the online programs. In addition, the former group also reported that the programs helped to improve their ability to cope with pandemic-related negative feelings (Table 2). In addition, the majority of adolescents who did continue to participate in online programs perceived their self-related health as “excellent”, a factor that is known to be correlated with psychological and physical health [38,39]. These findings provide support to the notion that such programs could play a pivotal role in empowering adolescents’ mental health and sense of support, safety and security.

Our next step was to assess the associations between program determinants and resilience. Adolescents who continue to participate in program activities during the lockdown period reported they perceived their sports program determinants as more meaningful compared to adolescents who discontinued their participation (Table 3).

We found that all program components (content of physical exercise, session initiation and session closure discussions, relationships with friends in the group, relationship with the group’s adult instructor and special program events) were positively correlated (Table 4). The highest positive correlation was found between relationships with friends in the group and relationships with the group’s adult instructor (r = 0.535, *p* < 0.001). Our multivariate analysis revealed that a positive relationship with the group’s adult instructor was the component of the program most strongly associated with resilience (Table 5). This is consistent with other studies that indicate that organized sports programs improve adolescents’ ability to cope with pandemic-related negative feelings and enhance resilience by providing an opportunity to develop close relationships and trust with the group instructor/guide [8,9]. Thus, similar to the results reported by Ruvalcaba et al. [20] that adolescents belonging to sports and social programs (e.g., Scouts) were more resilient, we conclude that participating in such programs can have positive effects on the ability of young people to cope with distress, even during adverse times like the present COVID-19 reality.

## 5. Limitations

Four notable limitations of this study should be mentioned. First, it is not a representative study as it used nonprobability sampling procedures and measuring. During the COVID-19 crisis in Israel, the public was restricted to a 100 m radius when leaving their home for nonessential activities. We, therefore, selected an online survey via social networks as the most suitable tool to enable us to reach a broad circle of participants in a short period of time. Indeed, our sociodemographic data suggest that our sample was diverse and included many adolescents enrolled in organized sports programs in 62 municipalities. Second, due to the study’s cross-sectional nature, it is difficult to make causal inferences, and the dynamic attitudes assessed in this study may change as the crisis progresses. The third limitation of this study is the fact that the study was conducted in Israel; as no parallel longitudinal study was conducted thus far in other countries, it is difficult to generalize the findings, especially considering varied biases and cultural diversities. Finally, the characteristics of the organized sports program questionnaire developed for this study were tested before only in a small pilot study. Further validation of this questionnaire in various populations (age groups, ethnicity and socioeconomic status) is required

## 6. Conclusions

The COVID-19 pandemic has forced the modern world to adapt rapidly to a new reality of physical and social distancing while introducing a range of new sources of stress and adversity. The results of our study demonstrate that the participation of adolescents in sports programs during the COVID-19 pandemic is associated with higher levels of resilience and physical activity and ability to cope, even though the activities were conducted online. A positive relationship with the group’s adult instructor and physical activity levels were associated with resilience. These findings are highly relevant and widely applicable since the possession of a well-rounded “tool-kit” of resilience-promoting factors is vital for strengthening adolescents’ resilience and avoiding negative outcomes, regardless of the difficulties they are facing. Thus, we recommend that to promote resilience and protect youth from negative health outcomes that are associated with adversity, uncertainty, and/or crisis, sports programs should continue their activities by any means available (even online meetings) while encouraging adolescents to participate.

## Figures and Tables

**Table 1:** Characteristics of the 473 adolescents who took part in the study, grouped by participation in online group meetings of organized sports programs during the COVID-19 lockdown.

	All *n* = 473	Did Not Participate *n* = 140	Participated *n* = 333	Diff between Groups
**Age (y)**	17.4 ± 0.8	17.3 ± 0.8	17.4 ± 0.8	Ns
**Gender (%)**				χ2(1) = 14.86 *p* < 0.001 *
*Male*	302 (63.8%)	71 (50.7%)	231 (69.4%)	
*Female*	171 (31.2%)	69 (49.3%)	102 (30.6%)	
*Grade*				NS
*10* *th*	71	50	21	
*11th*	169	55	114	
*12th*	202	60	142	
*Graduated*	31	4	32	
**Socioeconomic Status**				NS
*Not good at all*	2 (0.4%)	1 (0.7%)	1 (0.3%)	
*Not good*	12 (2.5%)	2 (1.4%)	10 (3.0%)	
*Average*	86 (18.2%)	27 (19.3%)	59 (17.8%)	
*Good*	217 (46.0%)	72 (51.4%)	145 (43.7%)	
*Very good*	155 (32.8%)	38 (27.1%)	117 (35.2%)	
**Born in Israel (%)**	441 (93.2%)	127 (90.7%)	314 (94.3%)	NS
**Religion (%)**				NS
*Secular*	376 (79.5%)	110 (78.6%)	266 (79.9%)	
*Traditional*	85 (18.0%)	27 (19.3%)	58 (17.4%)	
*Orthodox*	12 (2.5%)	3 (2.1%)	9 (2.7%)	

Abbreviations: NS, not significanct; * *p*-value of < 0.05 indicating statistical significance.

**Table 2:** Resilience, lifestyle, psychosocial and sports programs determinants of 473 adolescents during the COVID-19 lockdown grouped by participation in organized sports programs.

	ALL *n* = 473	Did Not Participate *n* = 140	Participated *n* = 333	Difference between Groups
**Resilience**	30.5 ± 5.6	29.3 ± 6.1	31.0 ± 5.3	T = 2.84 *p* = 0.005 *
**Physical activity**	227.3 ± 118.0	191.1 ± 145	242.5 ± 101.03	T = 4.41 *p* < 0.001
**Sleep (hours)**	8.2 ± 1.1	8.1 ± 1.2	8.3 ± 1.1	Ns
**Breakfast (%)**				χ2 = 14.89 *p* = 0.087 *
*Never*	29 (6.1%)	9 (6.4%)	20 (6.0%)	
*Less than 1 time/week*	26 (5.5%)	14 (10%)	12 (3.6%)	
*1–2 times/week*	60 (12.7%)	18 (12.9%)	42 (12.7%)	
*3–4 times/week*	70 (14.8%)	18 (12.9%)	52 (15.7%)	
*Every day or almost every day*	288 (60.8%)	82 (57.9%)	206 (62.0%)	
**Satisfaction with life**	6.9 ± 1.5	6.6 ± 1.7	7.0 ± 1.4	T = 2.43 *p* = 0.016 *
**Self Esteem**	24.0 ± 5.3	23.4 ± 5.4	24.3 ± 5.2	NS
**Self-Related Health (%)**				*p* = 0.006 *
*Poor*	3 (0.6%)	1 (0.7%)	2 (0.6%)	
*Fair*	12 (2.5%)	2 (1.4%)	10 (3%)	
*Good*	110 (23.3%)	47 (33.6%)	63 (18.9%)	
*Excellent*	348 (73.6%)	90 (64.3%)	258 (77.5%)	
**Number of close friends (%)**				*p* = 0.030 *
*Non*	6 (1.3%)	0 (0.0%)	6 (1.8%)	
*One*	10 (2.1%)	2 (1.4%)	8 (2.4%)	
*Two*	57 (12.1%)	12 (8.6%)	45 (13.5%)	
*Three or more*	400 (84.6%)	126 (90.0%)	274 (82.3%)	
**Loneliness (%)**				NS
*Seldom*	234 (49.5%)	73 (52.1%)	161 (48.3%)	
*Sometimes*	180 (38.1%)	52 (37.1%)	128 (38.4%)	
*Often*	44 (9.3%)	9 (6.4%)	35 (10.5%)	
*Very often*	15 (3.2%)	4 (3.4%)	9 (2.7%)	
**Morale (%)**				*p* = 0.085
*Very bad*	7 (1.5%)	1 (0.8%)	6 (1.8%)	
*Poor*	21 (4.4%)	4 (3.4%)	14 (4.2%)	
*Fair*	138 (29.2%)	40 (33.9%)	93 (27.9%)	
*Good*	223 (47.1%)	60 (50.8%)	151 (45.3%)	
*Very good*	84 (17.8%)	13 (11.0%)	69 (20.7%)	
**I feel that I am able to cope well during the COVID-19 Pandemic**				*p* = 0.034 *
*Strongly disagree*	1 (0.2%)	1 (0.7%)	0 (0.0%)	
*Disagree*	18 (3.8%)	4 (2.9%)	14 (4.2%)	
*Undecided*	65 (13.7%)	29 (20.7%)	36 (10.8%)	
*Agree*	197 (41.6%)	55 (39.3%)	142 (42.6%)	
*Strongly Agree*	192 (40.6%)	51 (36.4%)	141 (42.3%)	

Abbreviations: NS, not significanct; * *p*-value of < 0.05 indicating statistical significance.

**Table 3:** Assessment of how meaningful the sports program determinants are to adolescents during the COVID-19 lockdown grouped by participation in organized sports programs.

Program Determinant	ALL *n* = 363	Did Not Participate *n* = 104	Participated *n* = 259	Difference between Groups
Content of physical exercise	3.3 ± 1.2	2.2 ± 1.6	3.7 ± 0.6	*p* < 0.001
Session initiation and closure discussions	2.6 ± 1.4	1.3 ± 1.3	3.0 ± 2.6	*p* < 0.001
Relationships with friends in the group	3.2 ± 1.1	2.5 ± 1.6	3.5 ± 0.7	*p* < 0.001
Relationship with coach	3.1 ± 1.2	2.3 ± 1.6	3.4 ± 0.8	*p* < 0.001
Program special events	3.0 ± 1.3	2.0 ± 1.7	3.4 ± 0.9	*p* < 0.001

**Table 4:** Correlations between organized sports program components: content of physical exercise, session initiation and session closure discussions, relationships with friends in the group, relationship with the coach, and program special events.

Program Determinant Corr (r)	Content of Physical Exercise	Session Initiation and Closure Discussions	Relationships with Friends in the Group	Relationship with Coach	Program Special Events
Content of physical exercise	1				
Session initiation and closure discussions	0.442 **	1			
Relationships with friends in the group	0.291 **	0.287 **	1		
Relationship with coach	0.373 **	0.451 **	0.535 **	1	
Program special events	0.344 **	0.455 **	0.461 **	0.461 **	1

* Correlation is significant at the 0.05 level (2-tailed). ** Correlation is significant at the 0.01 level (2-tailed).

**Table 5:** Multiple regression analysis program components (physical activity, relationship with coach) and participant characteristics (age, gender, socioeconomic status) associated with resilience (primary outcome).

	β Coefficient	*p* Value
Age	1.141	0.001 *
Gender	2.853	<0.001 *
Socioeconomic status	1.249	0.029 *
Physical activity	0.008	<0.001 *
Relationship with coach	2.060	<0.001 *

Abbreviations: β Coefficient; Beta coefficient; * *p*-value of < 0.05 indicating statistical significance. Note: sports online program determinants that did not enter the regression equation: content of physical exercise, session initiation and session closure discussions, relationships with friends in the group, and program special events.

## Data Availability

The data presented in this study are available on request from the corresponding author, according to Ethics Committee of Tel-Aviv guidelines.

## Supplementary Materials

The following are available online at https://www.mdpi.com/article/10.3390/ijerph18084386/s1, Supplementary S1: Well-being during the Covid-19 pandemic among adolescents -Digital survey.

## References

[1] Zhai Y., Du X. (2020). Mental health care for international Chinese students affected by the COVID-19 outbreak. Lancet Psychiatry.

[2] Zarocostas J. (2020). How to fight an infodemic. Lancet.

[3] Brooks S.K., Webster R.K., Smith L.E., Woodland L., Wessely S., Greenberg N., Rubin G.J. (2020). The Psychological Impact of Quarantine and How to Reduce It: Rapid Review of the Evidence. Lancet.

[4] Read M.C. (2004). Effects of Quarantine. Emerg. Infect. Dis..

[5] Sanders C.E., Field T.M., Diego M., Kaplan M. (2000). Moderate involvement in sports is related to lower depression levels among adolescents. Adolescence.

[6] Scarf D., Moradi S., McGaw K., Hewitt J., Hayhurst J.G., Boyes M., Ruffman T., Hunter J.A. (2016). Somewhere I belong: Long-term increases in adolescents’ resilience are predicted by perceived belonging to the in-group. Br. J. Soc. Psychol..

[7] Ussher M.H., Owen C.G., Cook D.G., Whincup P.H. (2007). The relationship between physical activity, sedentary behaviour and psychological wellbeing among adolescents. Soc. Psychiatry Psychiatr. Epidemiol..

[8] Skrove M., Romundstad P., Indredavik M.S. (2013). Resilience, lifestyle and symptoms of anxiety and depression in adolescence: The Young-HUNT study. Soc. Psychiatry Psychiatr. Epidemiol..

[9] Zimmerman M.A., Stoddard S.A., Eisman A.B., Caldwell C.H., Aiyer S.M., Miller A. (2013). Adolescent Resilience: Resources and Assets for Informing Prevention. Child Dev. Perspect..

[10] Fergus S., Zimmerman M.A. (2005). Adolescent resilience: A Framework for Understanding Healthy Development in the Face of Risk. Annu. Rev. Public Health.

[11] Rutter M. (2012). Resilience as a dynamic concept. Dev. Psychopathol..

[12] Egeland B., Carlson E., Sroufe L.A. (1993). Resilience as process. Dev. Psychopathol..

[13] Gross J.J., Thompson R.A., Gross J.J. (2007). Emotion regulation: Conceptual foundations. Handbook of Emotion Regulation.

[14] Reker G.T., Wong. P.T.P., Birren J.E., Bengston V.L. (1988). Aging as an individual process: Toward a theory of personal meaning. Emergent Theories of Aging.

[15] Bonanno G.A., Pat-Horenczyk R., Noll J. (2011). Coping flexibility and trauma: The Perceived Ability to Cope with Trauma (PACT) scale. Psychol. Trauma Theory, Res. Pr. Policy.

[16] Ungar M. (2011). The Social Ecology of Resilience: A Handbook of Theory and Practice.

[17] Fletcher D., Sarkar M. (2013). Psychological Resilience: A Review and Critique of Definitions, Concepts, and Theory. Eur. Psychol..

[18] Dumont M., Provost M.A. (1999). Resilience in Adolescents: Protective Role of Social Support, Coping Strategies, Self-Esteem, and Social Activities on Experience of Stress and Depression. J. Youth Adolesc..

[19] Sippel L.M., Pietrzak R.H., Charney D.S., Mayes L.C., Southwick S.M. (2015). How Does Social Support Enhance Resilience in the Trauma-Exposed Individual?. Ecol. Soc..

[20] Ruvalcaba N.A., Gallegos J., Borges A., Gonzalez N. (2017). Extracurricular activities and group belonging as a protective factor in adolescence. Psicol. Educ..

[21] Chen P., Mao L., Nassis G.P., Harmer P., Ainsworth B.E., Li F. (2020). Coronavirus disease (COVID-19): The need to maintain regular physical activity while taking precautions. J. Sport Health Sci..

[22] Guthold R., Stevens G.A., Riley L.M., Bull F.C. (2020). Global trends in insufficient physical activity among adolescents: A pooled analysis of 298 population-based surveys with 1·6 million participants. Lancet Child Adolesc. Health.

[23] Baker R., Brick J., Bates N., Battaglia M., Couper M., Denver J., Gile K., Tourangeau R. (2013). Non-Probability Sampling. Rep. AAPOR Task Force Am. Assoc. Public Opin. Res. Boston MA.

[24] Heckathorn D.D. (2011). Comment: Snowball versus Respondent-Driven Sampling. Sociol. Methodol..

[25] Connor K.M., Davidson J.R.T. (2003). Development of a new resilience scale: The Connor-Davidson Resilience Scale (CD-RISC). Depress. Anxiety.

[26] Roberts C., Freeman J., Samdal O., Schnohr C.W., De Looze M.E., Nic Gabhainn S., Iannotti R., Rasmussen M., The International HBSC Study Group (2009). The Health Behaviour in School-aged Children (HBSC) study: Methodological developments and current tensions. Int. J. Public Health.

[27] Tesler R., Kolobov T., Ng K.W., Shapiro E., Walsh S.D., Shuval K., Harel-Fisch Y. (2019). Ethnic Disparities in Physical Activity among Adolescents in Israel. Am. J. Health Behav..

[28] Heatherton T.F., Wyland C.L. (2003). Assessing Self-Estee.

[29] Casale S., Flett G.L. (2020). Interpersonally-Based Fears during the COVID-19 Pandemic: Reflections on the Fear of Missing out and the Fear of Not Mattering Constructs. Clin. Neuropsychiatry.

[30] Danese A., Smith P., Chitsabesan P., Dubicka B. (2020). Child and adolescent mental health amidst emergencies and disasters. Br. J. Psychiatry.

[31] Zhou S.-J., Zhang L.-G., Wang L.-L., Guo Z.-C., Wang J.-Q., Chen J.-C., Liu M., Chen X., Chen J.-X. (2020). Prevalence and socio-demographic correlates of psychological health problems in Chinese adolescents during the outbreak of COVID-19. Eur. Child Adolesc. Psychiatry.

[32] Guan H., Okely A.D., Aguilar-Farias N., Cruz B.D.P., Draper C.E., El Hamdouchi A., Florindo A.A., Jáuregui A., Katzmarzyk P.T., Kontsevaya A. (2020). Promoting healthy movement behaviours among children during the COVID-19 pandemic. Lancet Child Adolesc. Health.

[33] Chen P., Mao L., Nassis G.P., Harmer P., Ainsworth B.E., Li F. (2020). Returning Chinese school-aged children and adolescents to physical activity in the wake of COVID-19: Actions and precautions. J. Sport Health Sci..

[34] https://www.who.int/ncds/prevention/physical-activity/factsheet_young_people/en/.

[35] Pearson N., Sherar L.B., Hamer M. (2019). Prevalence and Correlates of Meeting Sleep, Screen-Time, and Physical Activity Guidelines Among Adolescents in the United Kingdom. JAMA Pediatr..

[36] Sundar T.K.B., Løndal K., Lagerløv P., Glavin K., Helseth S., Galvin K. (2018). Overweight adolescents’ views on physical activity —Experiences of participants in an internet-based intervention: A qualitative study. BMC Public Health.

[37] Lippi G., Henry B.M., Sanchis-Gomar F. (2020). Physical inactivity and cardiovascular disease at the time of coronavirus disease 2019 (COVID-19). Eur. J. Prev. Cardiol..

[38] Fayers P.M., Sprangers M.A. (2002). Understanding self-rated health. Lancet.

[39] Wu S., Wang R., Zhao Y., Ma X., Wu M., Yan X., He J. (2013). The relationship between self-rated health and objective health status: A population-based study. BMC Public Health.

