# Supplementary material for: Continued Participation of Israeli Adolescents in Online Sports Programs during the COVID-19 Pandemic Is Associated with Higher Resilience
Source: Int J Environ Res Public Health. 2021 Apr 20;18(8):4386. doi: 10.3390/ijerph18084386 (PMC8074771; doi:10.3390/ijerph18084386)
Supplement: Supplementary file 1 [file ijerph-18-04386-s001.zip › ijerph-1167500-supplementary.pdf]

Appendix 1 – **Well-being during the Covid-19 pandemic among adolescents - Digital survey**

Participation in educational and physical activities

1. Do you participate in any kind of organized sports program?
   A. Yes
   B. No

2. If you do, what kind of physical activities do you engage in?
   A. Competitive sports
   B. Dance
   C. Hamesh Ezbaot Organization
   D. Martial arts
   E. Gym
   F. Other ____
   G. I do not participate in any kind of physical activities

3. Has your sports group or organization remained active during the current pandemic crisis?
   A. Yes
   B. No

4. How long has it been since you joined your sports group or organization?
   A. Month to three months
   B. Four to six months
   C. Six to twelve months
   D. More than a year
   E. I am not a member of any kind of sports group or organization

5. Are you a member of any kind of youth movement?
   A. Yes
   B. No

6. If you are a member, which of the following youth movements do you belong to?
   A. HaTzofim (Hebrew Scouts)
   B. Knafaim shel Krembo (Krembo Wings, a Youth Movement for Children with and without Disabilities)
   C. HaNoar HaOved (Federation of Young Students and Workers)
   D. HaShomer haTzair (The Young Guard)
   E. Bney haMoshavim
   F. Bney Akiva
   G. Other ____
   H. I am not a member of any kind of youth movement

7. How long has it been since you joined your youth movement?
   A. Month to three months
   B. Four to six months
   C. Six to twelve months
   D. More than a year
   E. I am not a member of any kind of youth movement

<u>The following questions have to do with your sports group or organization</u>

8. Where do the activities take place? ____

9. How often do you participate in the regular meetings of your group or organization?
   A. I rarely participate
   B. I participate irregularly
   C. I usually participate
   D. I always participate

10. On average, how many times a month do you exercise?
    ____

11. What is the main reason why you participate in the activities?
    A. To improve my fitness levels
    B. To prepare for my army service
    C. To improve my self-confidence
    D. To get together with friends
    E. To gain leadership and excellence skills
    F. To improve my health
    G. To learn and grow
    H. To help others
    I. Other

Grade how important to you are the following factors in the group or organization that you exercise with:

	1	2	3	4	5
	Not at all important	Slightly important	Somewhat Important	Moderately important	Very important
12. content of physical exercise					

13. session initiation and closure discussions					
14. relationship with the instructor or guide					
15. relationships with friends in the group					
16. program special events					

17. If any other significant factors came to your mind, please share: ______

18. How would you define yourself when it comes to exercise. How much do you like to exercise:
    A. Not at all
    B. Slightly
    C. Moderate
    D. To a large extent
    E. To a very large extent

19. In general, how many times do you usually engage in strenuous exercise in your free time?
    A. Every day
    B. 4-6 times a week
    C. 2-3 times a week
    D. Once a week
    E. Once a month
    F. Less than once a month
    G. Never

20. During the last 7 days, in how many days did you exercise that lasted a total of at least 60 minutes a day?
    A. 0 days
    B. One day
    C. Two days
    D. 3 days
    E. 4 days

F. 5 days
G. 6 days
H. 7 days
